# Data on the cooling rate using nano carbon-fluid quenching medium and its effect on the hardness of S45C steel

**DOI:** 10.1016/j.dib.2022.108867

**Published:** 2022-12-30

**Authors:** Ramzul Irham Riza, Kholqillah Ardhian Ilman

**Affiliations:** Department of Mechanical Engineering, Faculty of Engineering, Universitas Muhammadiyah Surakarta, Indonesia

**Keywords:** Nanoparticle, Nanofluid, Heat treatment, Quenching, Thermal conductivity, Cooling rate

## Abstract

This dataset provides the effect of cooling rate using nano-fluid quenchant. The nanofluid consists of 0.1, 0.3, and 0.5 gram of nano-sized carbon particles in 100 ml of distilled water. Pure distilled water was also used as a comparison control. The particle was produced by using planetary ball-mill at 500 rpm for 15 hours. Field-Emission Scanning Microscope (FE-SEM) and Energy Dispersive X-Ray (EDX) were also used to confirm the size, shape, and purity of the nanoparticles. These nanofluids were then applied to quench annealed S45C carbon steel samples. The samples were connected to a thermocouple with a temperature data logger to observe the cooling rate of the nano-fluid quenchant. The quenched samples were tested to get the information on the hardness of S45C carbon steel.

**Specifications Table**

**Table:** 

Subject	Material Science
Specific subject area	Quenching effect on metal hardness
Type of data	Table in excel format and original testing report (PDF format)
How data were acquired	Instruments: Thermometer AZ Instrument 88598 (4 Channel K Thermometer SD Logger), Field-Emission Scanning Microscope, Energy Dispersive X-Ray, Vickers and Rockwell Hardness Testing Machine
Data format	Raw
Parameters for data collection	Planetary ball-mill was used at 500 rpm for 15 hours to produce nano-sized carbon particle followed by making nanofluid using the nanoparticle. The nanofluid consists of 0.1, 0.3, and 0.5 gram of nano-sized carbon particles in 100 ml of distilled water. Data on cooling rate was recorded using a temperature data logger connected to the surface while it is on quenching process. After the quenching process, data on the hardness value of S45C steel was collected.
Description for data collection	Information on the cooling rate of the quenched S45C steel using nanofluid media. The data of temperature change was obtained using a data logger connected to a thermocouple attached to the specimen surface. Temperature changes are recorded every 1 second. Hardness data of the specimens was taken after the quenching process using the method of Rockwell and Vickers test.
Data source location	Universitas Muhammadiyah Surakarta, Indonesia
Data accessibility	Repository name: Mendeley Data Data identification number: DOI: 10.17632/5z9h2wj8zm.2 Direct URL to Data: https://doi.org/10.17632/5z9h2wj8zm.2

## Value of the Data

• The raw data may be used for more investigation on material science and heat treatment to modify the mechanical properties of metals.
• The raw data will be useful to those involved in the manufacturing of metal-based products and components.
• The data can be re-used as a comparison of various effects of different content of nanoparticles, especially in relation to the quenching process of carbon steel.

## 1 Data Description

This article comprises three datasets: Cooling rate of different nanoparticle contents, report of material testing, and testing report of hardness value. The data of cooling rates are described in four following columns: time (in hours: minutes: seconds format), interval (in second), recorded temperature, and degree (in Celcius). Temperature changes were collected using Thermometer AZ Instrument 88598 (4 Channel K Thermometer SD Logger). A report of material testing is provided in PDF format to inform the content of elements in the carbon steel (S45C). Data on hardness value of the steel is also presented (in HVN and HRc). The files for the datasets are in table (in .xlsx), graph, and original testing report (PDF format). Table 1 lists the description for every file.

**Table 1:** Description of each given file.

File Name	Description
0 gr - nano particle.xlsx	Cooling rate data of 0 gr nanocarbon fluid in 100 ml of distilled water
0.1 gr - nano particle.xlsx	Cooling rate data of 0.1 gr nanocarbon fluid in 100 ml of distilled water
0.3 gr - nano particle.xlsx	Cooling rate data of 0.3 gr nanocarbon fluid in 100 ml of distilled water
0.5 gr - nano particle.xlsx	Cooling rate data of 0.5 gr nanocarbon fluid in 100 ml of distilled water
Report of Material Testing.pdf	Content of elements in the S45C carbon steel
Rockwell and Vickers -Testing Report.pdf	Data on hardness Vickers Number (HVN) and Rockwell C (HRc)

## 2 Experimental Design, Materials and Methods

This study consists of three main phases [1]. The first step was making specimens of S45C steel by 10 mm diameter and 10 mm of thickness as 4 pieces. Second stage was preparation of nanofluid solution by using planetary ball milling machine for 15 hours of processing to get 561nm carbon particles and mixing them with distilled water by using ultrasonic machine for 15 minutes. Field-Emission Scanning Microscope (FE-SEM) and Energy Dispersive X-Ray (EDX) were utilized to confirm the size, shape, and purity of the nanoparticles. Compositions of nanocarbon particle are: 0 gram, 0.1 gram, 0.3 gram and 0.5 gram in 100 mL of distilled water.

The process began by heating the samples and holding it for one hour at a temperature around 1000°C. After that, the sample was quenched using a carbon nano solution with the following carbon composition: 0 gram, 0.1 gram, 0.3 gram, and 0.5 gram. Data on cooling rate was obtained in the process of quenching using thermocouple with data logger connected to the sample surface. After the quenching process, data on the hardness value of S45C steel was collected using Vickers and Rockwell methods.

## CRediT authorship contribution statement

**Ramzul Irham Riza:** Conceptualization, Methodology, Supervision, Investigation, Writing – original draft. **Kholqillah Ardhian Ilman:** Data curation, Validation, Writing – review & editing.

## Declaration of Competing Interest

The authors certify that they have no conflict in financial interests or personal relations which have impacted the work described in this paper.

## Data Availability

Data on the cooling rate using nano carbon-fluid quenching medium and its effect on the hardness of S45C steel (Original data) (Mendeley Data).
